# Experimental Study on Characteristics of Grinded Graphene Nanofluids with Surfactants

**DOI:** 10.3390/ma11060950

**Published:** 2018-06-04

**Authors:** HeonJin Seong, GwiNam Kim, JongHoon Jeon, HyoMin Jeong, JungPil Noh, YoungJu Kim, HyunJi Kim, SunChul Huh

**Affiliations:** 1 Department of Energy and Mechanical Engineering, Gyeongsang National University, 38, Cheondaegukchi-gil 53064, Tongyeong-si, Korea; veruzurya@naver.com (H.S.); wwgcw1480@naver.com (J.J); hmjeong@gnu.ac.kr (H.J.); nohjp@gnu.ac.kr (J.N.); 2 Department of Mechanical & Automotive Engineering, Suncheon Jeil College, 17 Jeildaehak-gil 57997, Suncheon-si, Korea; rnlska0717@nate.com; 3 Department of Exploration System Research, KIGAM Pohang Branch, 905, Yeongilman-daero 37559, Pohang-si, Korea; kyjp7272@kigam.re.kr (Y.K.); hjkigam@kigam.re.kr (H.K.)

**Keywords:** graphene, thermal conductivity, nanofluid, surfactant, zeta potential, dispersibility

## Abstract

In earlier studies, much research has focused on increasing the efficiency of heat exchanger fields. Therefore, in this study, graphene nanofluid was fabricated for use as a heat transfer medium for a heat exchanger. Graphene has excellent electrical conductivity, mechanical properties, and heat transfer properties. It is expected that the heat transfer efficiency will be improved by fabricating the nanofluid. However, graphene is prone to sedimentation, because of its cohesion due to van der Waals binding force. In this experiment, a nanofluid was fabricated with enhanced dispersibility by surfactant and the ball-milling process. The zeta potential, absorbance, and thermal conductivity of the nanofluid were measured. As a result, when using the ratio of 2:1 (graphene:sodium dodecyl sulfate (SDS)), a higher thermal conductivity was obtained than in other conditions.

## 1. Introduction

As the industry has developed, heat exchangers have been increasingly used in a variety of fields, including food, cooling and heating, shipbuilding, and chemistry. Thus, if the efficiency of the heat exchanger can be increased, the energy consumed and the generation of carbon dioxide can both be reduced [1,2]. Common fluids in commercial applications that are used in heat exchangers (including water, ethylene glycol, and oil) usually have a low thermal conductivity; as a result, many attempts have been made to improve the heat transfer rate of these fluids. As an example, the addition of nanoparticles can be cited [3,4]. Adding nanoparticles creates a mixture called nanofluid and improves the heat transfer coefficient [5,6]. Choi [7] first introduced the concept of nanofluids produced by mixing nanoparticles with a relatively higher thermal conductivity than conventional heat transfer media in order to increase the efficiency of the heat exchanger. Nanofluid is a new concept of heat transfer fluid in which nano-sized solid particles with excellent thermal conductivity are stably suspended in a pure fluid that has excellent thermal properties and is expected to be a next-generation heat exchange medium [8,9]. Considering the main idea of adding nanoparticles to conventional fluids, i.e., enhancing the heat transfer performance of the working fluid by making improvements in thermal conductivity, massive research has been dedicated to introducing new materials with super thermal conductivity properties [10,11]. Among all of the propositions, a higher thermal conductivity and lower density of carbon materials compared with metals and metal oxides have made nanofluids the most attractive substances [12]. In this regard, many investigations have been carried out to study the properties of various structural forms of carbon nanomaterials [13,14,15] such as carbon nanotubes [16,17], graphite nanoparticles [18], and diamond nanoparticles [19]. Among the various nanoparticles added to nanofluids, graphene, which is reported to exhibit thermal conductivity as high as 5300 W/mK, is attracting attention [20,21]. It is because among the four outermost electrons in the carbon atom, three electrons form a sigma-bond to form a hexagonal structure, and a long range of pi conjugates with the one remaining electron [22]. However, since graphene is susceptible to flocculation and precipitation due to van der Waals binding force, the technique of securing dispersion stability and fabricating nanofluid is the most important problem in utilizing graphene nanofluid as a heat transfer medium. Previous graphenes were grinded using a planetary ball mill instrument according to various conditions, and graphene was pulverized under the condition that the thermal conductivity and the dispersion were the highest in the study of thermal conductivity [23,24]. After preparing the nanofluid based on distilled water, surfactant (SDS, SDBS) [25,26,27] was added at each ratio and dispersed by using an ultrasonic exciter due to the negatively charged carbon-based nanomaterial and anionic surfactants, which have the presence of electrostatic repulsion between them. The zeta potential and thermal conductivity measurements were used to measure the dispersion stability and thermal conductivity, respectively. The purpose of this study is to prepare nanofluids that stably suspend and disperse graphene using a surfactant.

## 2. Materials and Methods

### 2.1. Nanofluid Preparation

In this study, DW (distilled water) was used for the base fluids that were produced through the membrane-type DW maker, which maintained a water quality under 10 ppm for the total dissolved solids (TDS). Graphene with 7-nm thickness and a 40-nm size with a specific surface area of 100 m^2^/g and purity of 99.9% was purchased from Graphene supermarket, and surfactant SDS and SDBS were purchased from Junsei Chemical Co., Ltd. (Tokyo, Japan) (SDS), TOKYO Chemical Industry Co., Ltd. (Tokyo, Japan) (SDBS) was used. First, 0.1 g of graphene was pulverized with a ball size of 1 mm, a ball-milling speed of 200 rpm, and a ball-milling time of 60 min using (a) the planetary ball mill instrument. After that, the ratio of graphene (0.1 wt %) dissolved in the water and the surfactant SDS and SDBS were prepared for adding in ratios of 1:3, 1:2, 1:1, 2:1, and 3:1. Furthermore, ultrasound excitation was carried out for 40 min to make the graphene nanofluids, and the degrees of nanoparticle dispersion were measured by using UV spectroscopy.

### 2.2. Measuring Equipments

**Figure 1:**
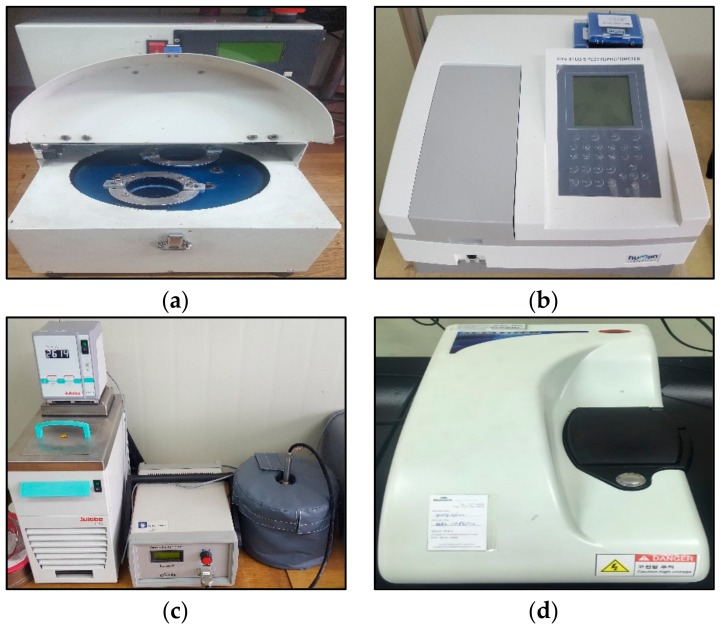
Photograph of measurement equipment: (**a**) planetary ball mill; (**b**) UV-vis spectrophotometer; (**c**) thermal Conductivity measuring system LAMBDA; (**d**) Zetasizer Nano ZS.

The equipment used in this experiment is shown in Figure 1, and included (a) a planetary ball mill instrument used to grind graphene. In addition, the absorbance of the prepared nanofluids was measured using (b) the UV-vis spectrophotometer, while the thermal conductivity was measured using (c) a LAMBDA instrument, and the dispersion stability was determined by measuring the zeta potential with (d) a Zetasizer nano ZS.

### 2.3. Measurements Procedure of the Thermal Conductivity

**Figure 2:**
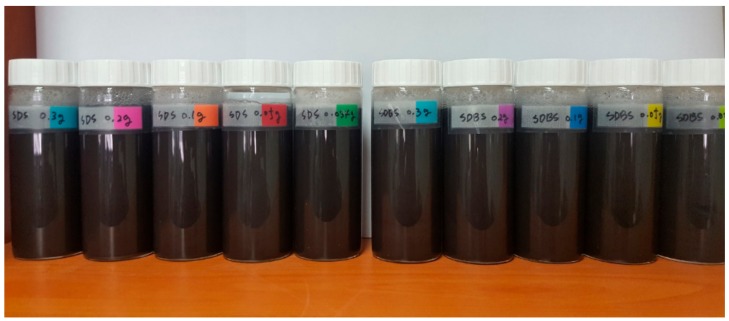
Sample of graphene nanofluids + sodium dodecyl sulfate (SDS) (left)/+ SDBS (right).

The thermal conductivity measuring system LAMBDA in Figure 1c of this experiment measured the nanofluid of Figure 2 based on the transient hot-wire method. The LAMBDA system is composed of three components: the measuring head, the microprocessor unit for control and evaluation, and the software-controlled heating/cooling device. A platinum wire with a 0.1-mm diameter was employed as the hot wire in the measuring head. This wire served as both the heating unit and thermometer, as there is a linear relationship between the electrical resistance and the temperature of the wire. The detailed principles of this have been introduced in previous studies [28].

## 3. Results and Discussion

**Figure 3:**
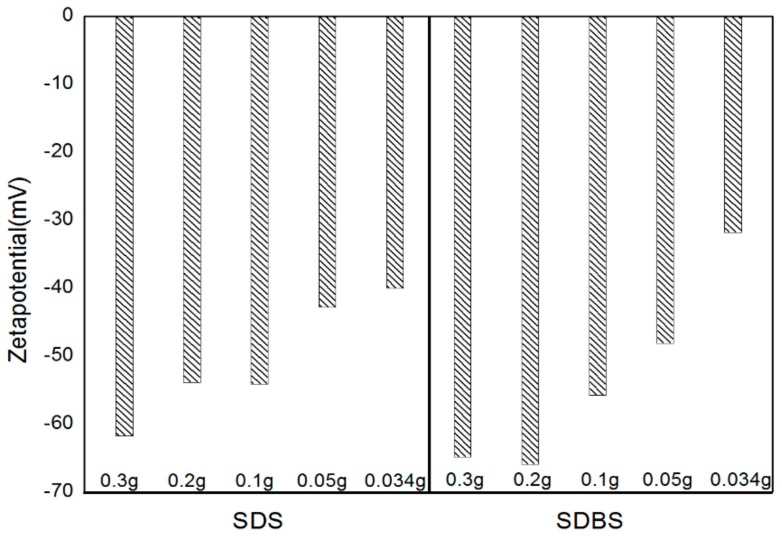
Zeta potential of graphene by various conditions.

A graph of the zeta potential, which was measured using a ZetaSizer Nano ZS instrument, is shown in Figure 3. The zeta potential is a measure of the repulsive force and attraction force between the nanoparticles suspending in the liquid [29], and the relative stability of dispersion can be confirmed by the magnitude of the zeta potential value. The closer the zeta potential is to 0 mV, the higher the degree of aggregation, and the higher the absolute value, the higher the dispersion stability [30]. For instance, nanoparticles in the dispersion are stable when the absolute value of the zeta potential is higher than ±30 mV [31]. SDS showed the highest zeta potential value when 0.3 g was added, and lowest value when 0.034 g was added. SDBS showed a similar tendency, and the absolute value of the zeta potential was higher than that of SDS when SDBS was added. Even if the error range in the experimental measurement was taken into consideration, the absolute value of the zeta potential increased. Furthermore, the dispersion stability was high as the amount of the surfactant was increased in the case of under the critical micelle concentration (CMC) of the surfactant [32].

**Figure 4:**
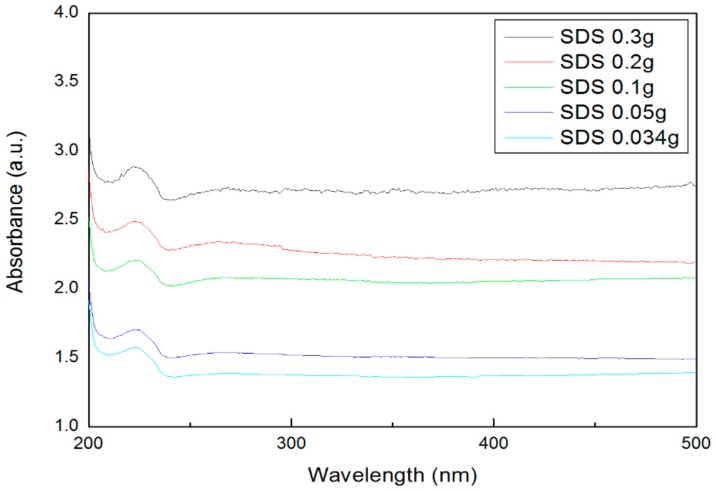
Absorbance of graphene by SDS addition ratio.

**Figure 5:**
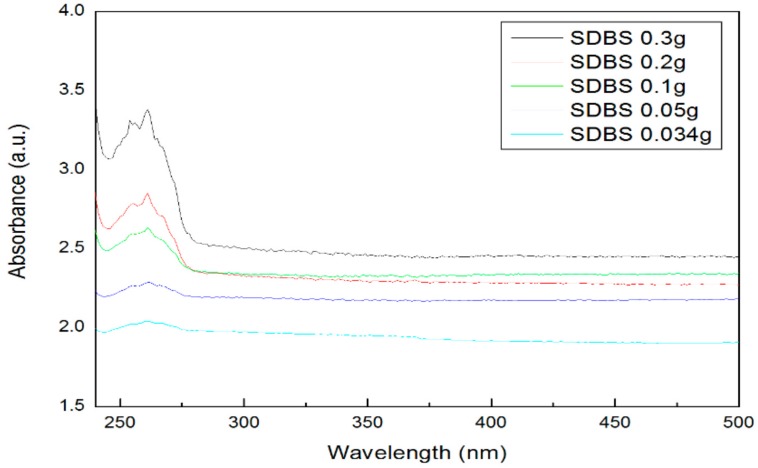
Absorbance of graphene by SDBS addition ratio.

Figure 4 shows an absorbance graph according to the addition amount of the surfactant SDS. The main peak was observed at a wavelength of about 230 nm. The highest absorbance was found at 0.3 g, according to the addition amount of SDS, and the lowest absorbance was found at 0.034 g. Figure 5 shows the absorbance according to the addition amount of SDBS. The main peak is shown near the wavelength of 260 nm, and as shown in Figure 5, the highest absorbance was obtained at 0.3 g, which was the highest amount of SDBS added, and the lowest absorbance was obtained at 0.034 g of SDBS. It can be seen that the reason for the main peak differences between SDS and SDBS depended on the surfactant containing benzene. The absorbance results showed a similar tendency compared with the zeta potential, and it was confirmed that the dispersibility increases as the surfactant amount increases.

**Figure 6:**
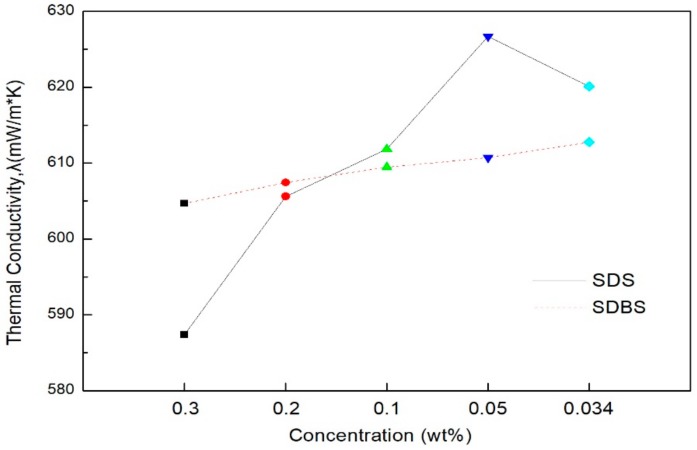
The mean thermal conductivity of graphene nanofluid according to the amount of surfactant.

The mean thermal conductivity of the graphene nanofluids according to the amount of surfactant added is shown in Figure 6. Overall, the mean thermal conductivity of surfactant SDS shows greater differences than the mean thermal conductivity of surfactant SDBS. When the addition amount of the surfactant was 0.3 g and 0.2 g, SDS showed a lower thermal conductivity than SDBS, but when the addition amount was lower than 0.1 g, SDS showed a higher thermal conductivity than SDBS. This suggests that when the addition amount of surfactant SDS was 0.1 g or more, the heat transfer of the graphene nanoparticles was suppressed. In addition, the lower the amount of surfactant added, the higher the thermal conductivity; when the addition amount of surfactant SDS was 0.05 g, the highest thermal conductivity was shown among all of the conditions.

**Figure 7:**
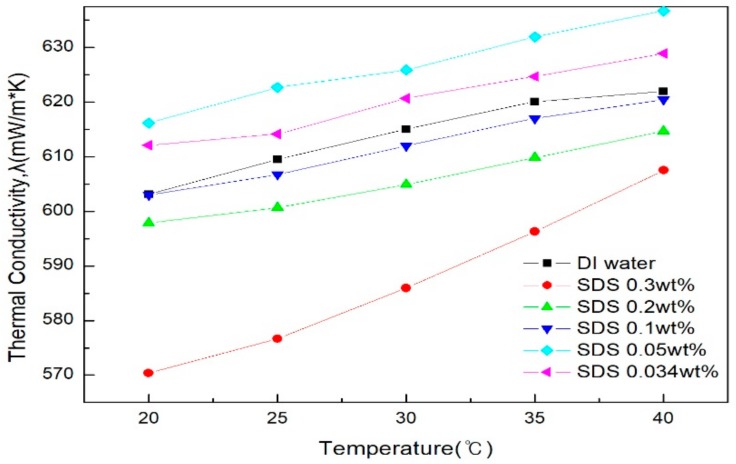
Thermal conductivity of graphene by SDS addition ratio.

**Figure 8:**
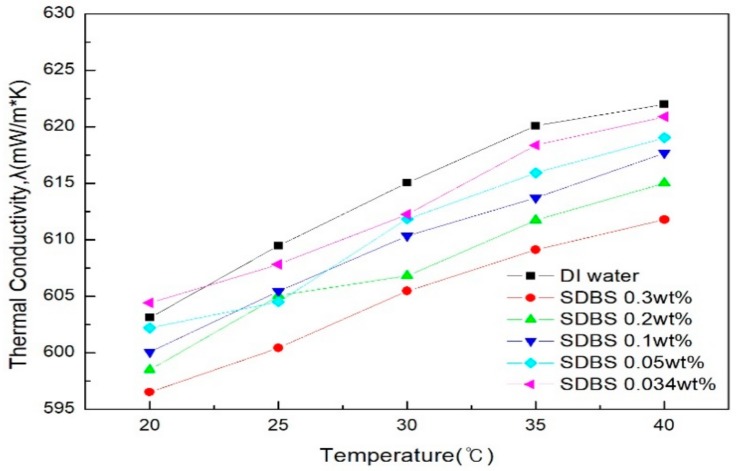
Thermal conductivity of graphene by SDBS addition ratio.

Figure 7 and Figure 8 show the thermal conductivity graphs of graphene by SDS and SDBS addition ratio, as measured in comparison with distilled water. Figure 7 shows the result of SDS; here, the thermal conductivity is higher than that of distilled water when the addition amount is 0.05 g and 0.034 g, and the thermal conductivity shown in Figure 8 for SDBS is lower than that of distilled water in all of the conditions. It can be analyzed that when the amount of SDS is lower than the amount of graphene, it has great thermal conductivity. In addition, it was confirmed that the higher the addition ratio of the surfactant as a whole, the lower the thermal conductivity. This indicates that the addition of a large amount of surfactant may increase the absorbance and dispersion stability of the graphene nanofluid, and conversely, the improvement in thermal conductivity was suppressed [33]. When SDS was added as a whole, the thermal conductivity was higher than that of SDBS, and the results were similar to those of Kim et al. [34]. In addition, the highest thermal conductivity was obtained when the ratio of graphene to SDS was 2:1 (SDS 0.05 g), and further studies on this addition ratio would be needed.

## 4. Conclusions

In this study, graphene was grinded using a planetary ball mill instrument to stably disperse and the graphene nanofluid; (0.1 wt %) graphene nanofluid was prepared by adding surfactant to each condition. In order to measure the dispersion and dispersion stability, the zeta potential and the absorbance through UV were measured. The thermal conductivity was measured using the transient hot-wire method, and the following conclusions were obtained.

(1) The absorbance was measured to confirm the dispersibility. The highest absorbance was obtained when 0.3 g of SDS and SDBS were used, and the lowest absorbance was obtained when 0.034 g of SDS and SDBS were used. As a result, as the surfactant ratio increases, the nanofluid containing SDS and SDBS produced higher absorbance values.
(2) The zeta potential was measured to figure out the dispersion stability. Absolute values of 30 mV or more were obtained under all of the conditions, and it could be judged that the value of the dispersion stability was sufficiently high. In this experiment, SDBS showed that the zeta potential value was higher than SDS, and had a higher dispersion stability as the amount of surfactant was increased.
(3) The thermal conductivity was measured in comparison with distilled water. Graphene nanofluid with SDS added showed a higher thermal conductivity than when SDBS was added. SDS showed a higher thermal conductivity than distilled water at the ratio of graphene to surfactant of 2:1 (0.05 g) and 3:1 (0.034 g) and generally, SDBS showed a lower thermal conductivity than distilled water at all of the conditions. When the mean thermal conductivity of the two surfactants was compared, SDS showed a higher increase in thermal conductivity than the SDBS as the amount of surfactant decreased. When the ratio of graphene to SDS was 2:1 (0.05 g), it showed the highest thermal conductivity. As a result of thermal conductivity measurement, it was confirmed that the thermal conductivity becomes smaller according to the amount of the surfactant.
